# Exploring the determinants influencing suicidal ideation and depression in gastrointestinal cancer patients

**DOI:** 10.1038/s41598-023-45634-x

**Published:** 2023-10-25

**Authors:** Avishek Choudhury, Yeganeh Shahsavar

**Affiliations:** Industrial and Management Systems Engineering, Benjamin M. Statler College of Engineering and Mineral Resources, West Virginia University, 1306 Evansdale Drive, Morgantown, WV 26506 USA

**Keywords:** Cancer, Gastroenterology, Health care, Psychology and behaviour

## Abstract

Studies have shown a heightened prevalence of depression and suicidal ideation among patients with Gastrointestinal Cancer (GIC). GIC patients are at a 1.5- to threefold increased risk of suicide and depression compared to other cancer patients. This study investigates the interplay of internet use, family burden, and emotional support on mental health (depression) and suicidal ideation among patients with GIC. The study involves 202 respondents of which 78 were undergoing GIC treatment during this study. Using structural equation modeling, our findings indicate a substantial negative correlation between mental health and suicidal ideation. Overall, suicidal ideation (median score) was noticeably lower in patient who completed their treatment with noticeable individuals with exceptionally high SI even after completing the treatment. Notably, participants who had completed their treatment demonstrated a significantly stronger correlation between emotional support and mental health compared to those who were still undergoing treatment. Age was found to moderate the mental health-suicidal ideation link significantly. Internet usage for health-related information was also inversely correlated with mental health (directly) and suicidal ideation (indirectly). We noted that the influence of emotional support on mental health was significantly higher among individuals who completed their treatment compared to those who were undergoing their GIC treatment. Family burden emerged as significant negative influences on mental health, while emotional support positively impacted mental health. The findings of this study contribute towards a deeper understanding of suicide risk factors in GIC patients, potentially shaping more effective preventive strategies.

## Introduction

Gastrointestinal cancer (GIC) refers to a group of cancers that affect the gastrointestinal tract and organs involved in digestion. The main types of GIC include esophageal cancer, gastric (stomach) cancer, liver cancer, pancreatic cancer, gallbladder and bile duct cancer, small intestine cancer, colorectal cancer, and anal cancer. These cancers constitute a major public health concern due to their global prevalence, morbidity, and mortality rates. The ripple effect of GIC extends beyond individual patient diagnoses, impacting families, communities, and healthcare systems.

Alongside the physical health repercussions, there's an increasing acknowledgment of the psychological toll these cancers can exact^1–4^. Studies have shown a heightened prevalence of depression and suicidal ideation among GIC patients^5–11^. GIC patients are at a 1.5- to threefold increased risk of suicide and depression compared to other cancer patients^12,13^. A population-based study on GIC patients found that the suicide rate was highest within the first three months following a cancer diagnosis^14^. Studies have acknowledged loneliness, gender, income, marital status, cancer stage, comorbidity, quality of life, and social support as factors influencing depression and suicide risk in this population^5,15–18^.

**Figure 1:**
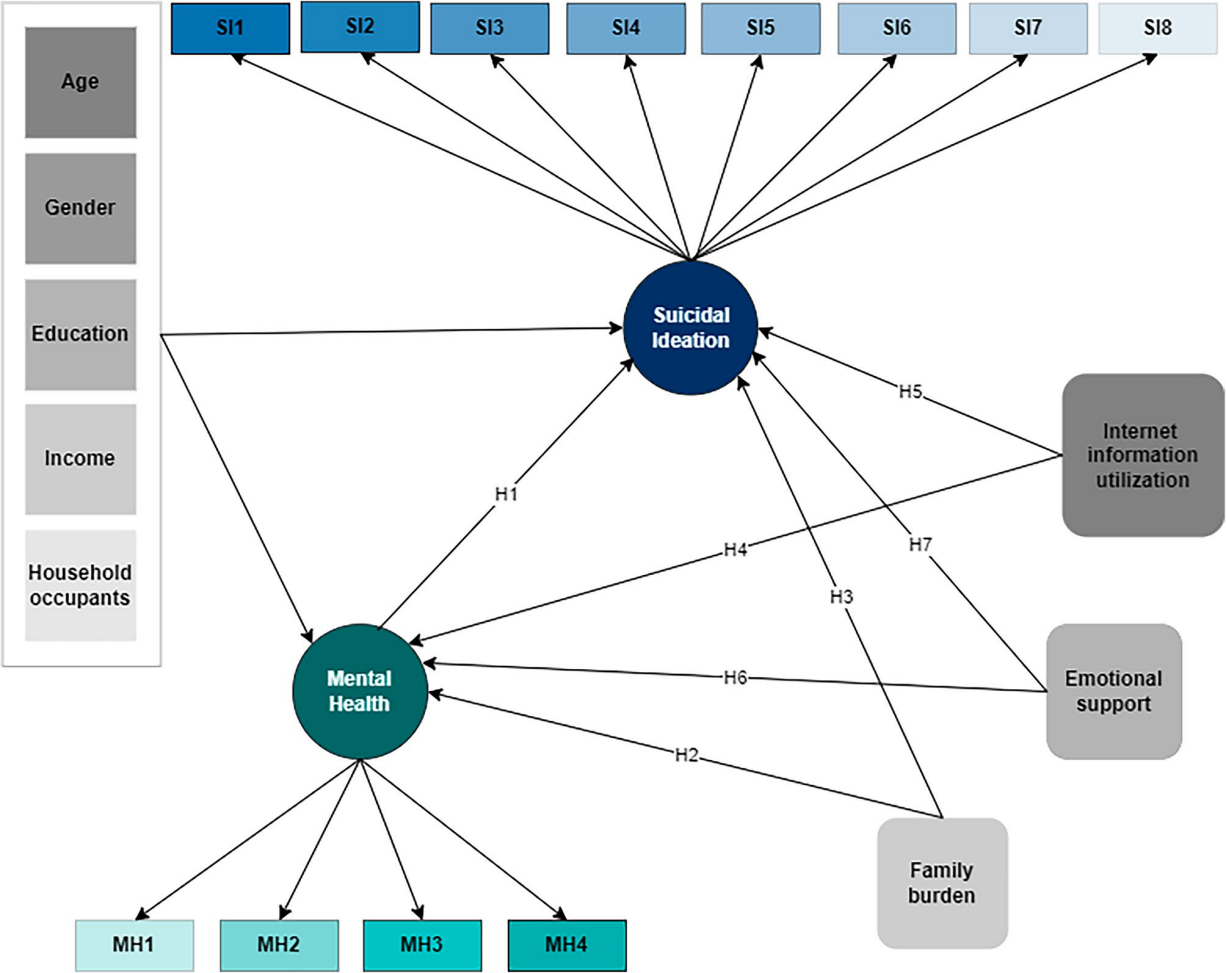
Conceptual framework illustrating the relationships between ‘Suicidal Ideation,’ ‘Mental health (inverse measure of depression),’ ‘Family burden,’ ‘Internet information utilization,’ ‘Emotional support,’ ‘Age,’ ‘Gender,’ ‘Income,’ ‘Education,’ and ‘Household occupants.’

Mental health-related problems such as depression, anxiety, and suicidal ideation (SI) are also substantial across other cancer types, including breast cancer^19^, bladder cancer^20^, lung cancer^21^, and head and neck cancer^22^ where economic burden, time since diagnosis, surgical treatment, and nutrition play a major role^23,24^. The current body of research has shed some light on the psychological implications of GIC. Yet, it falls short of exploring the complex association of (a) internet use, (b) family burden, and (c) emotional support on depression and suicidal ideation in this patients population. In this paper, we extend the literature by exploring the impact of internet information use, family burden, emotional support, age, gender, education, income, household occupants, mental health (depression), and suicidal ideation of patients with GIC, as shown in Fig. 1.

### Internet information utilization

The advent of the digital age, characterized by the proliferation of online resources and extensive internet connectivity, has fundamentally altered patients' interaction with healthcare information and services. Patients diagnosed with cancers now can consult the internet for various reasons, ranging from understanding their diagnosis and potential treatment options to seeking solace in online support groups. Despite this broadening digital health landscape, our understanding of the internet's influence on patients remains limited, particularly in relation to GIC patients. As we delve into the digital age, the internet's 'double-edged sword' nature becomes apparent^25^. On the one hand, patients can gain substantial insight into their condition from reputable medical sources. For example, a newly diagnosed pancreatic cancer patient may find reliable information on treatment options, survival rates, and lifestyle adjustments, which can bring a sense of control and reduce anxiety. However, on the flip side, they could stumble upon misleading information that can exacerbate their worries^25^. Suppose the same patient comes across a website claiming an extremely low survival rate without clarifying that it refers to late-stage cases; the patient may misinterpret this as a blanket statement, inducing panic and despair.

Internet use may contribute to the development of cyberchondria, a condition characterized by increased health anxiety resulting from online health information-seeking behavior^26^. A study found that the use of the internet for health information can lead to heightened anxiety and depression among cancer patients, particularly when they encounter conflicting or ambiguous information^27^. This is especially concerning for GIC patients, who might already be grappling with the physical and emotional toll of their diagnosis and treatment. Evidence also associates internet use with an increased likelihood of suicidal ideation where a study showed that the isolation and negative emotions associated with excessive internet use can exacerbate feelings of despair and hopelessness, which are known risk factors for suicidal ideation^28^. Given the unique challenges faced by GIC patients, it becomes crucial to understand how internet use for health information impacts them.

### Family burden

It refers to the emotional, physical, and financial challenges faced by the family members of individuals with chronic illnesses, including cancer^29^. It can be caused by a number of factors, including the diagnosis of cancer itself, the side effects of treatment, the disruption of social and family life, and the financial costs of care^29^. A study found that cancer patients who reported high levels of family burden were more likely to experience depression and anxiety than cancer patients who reported low levels of family burden^30^. Similarly, research has also shown that increased family burden can lead to psychological distress, which in turn may result in suicidal thoughts and behaviors among cancer patients^31^. Liu et al.^32^ identified self-perceived burden as a crucial factor associated with suicidal ideation in cancer patients, with family burden playing a significant role in contributing to this self-perception.

### Emotional support

In the context of this study, emotional support is defined as the care, empathy, encouragement, and affirmation provided by close ones, encompassing family, friends, and medical professionals, which aids individuals in coping with their health condition. Research has shown that emotional support is strongly associated with mental health and suicidal ideation in cancer patients. Cancer patients who receive emotional support and psychological assistance are less likely to experience depression and suicidal ideation^33^. Emotional support from family and friends has been identified as the most important source of support for cancer patients^34^. In addition, a strong therapeutic alliance between patients and their oncologists has been found to reduce the risk of suicidal ideation^35^. Conversely, cancer patients who lack emotional support are at a higher risk of experiencing suicidal ideation^36,37^.

In the context of this study, it's important to clarify that our usage of the term 'mental health' focuses on the assessment of depressive symptoms. In other words, it is an inverse measure of depression. While we acknowledge that the broader concept of mental health encompasses a wide range of positive and negative dimensions, our research instruments were specifically oriented towards capturing the prevalence and intensity of depression among the participants. We particularly explore the following hypotheses:

- Hypothesis 1 (H1): Mental health is inversely correlated with the prevalence of suicidal ideation in patients diagnosed with GIC.
- Hypothesis 2 (H2): Family burden inversely correlates with the mental health of patients diagnosed with GIC.
- Hypothesis 3 (H3): Family burden positively correlates with the incidence of suicidal ideation among patients with GIC.
- Hypothesis 4 (H4): Internet information utilization for health-related information inversely correlates with the mental health of patients with GIC, possibly due to exposure to misinformation or misinterpretation.
- Hypothesis 5 (H5): Internet information utilization for health-related information positively correlates with the development of suicidal ideation in patients with GIC.
- Hypothesis 6 (H6): Emotional support positively correlates with the mental health of patients with GIC.
- Hypothesis 7 (H7): Emotional support inversely correlates with the prevalence of suicidal ideation in patients with GIC.

## Methods

### Ethics statement

The research received approval from the Institutional Review Board at West Virginia University in Morgantown, West Virginia, USA (Protocol Number 2212691613). The study was performed in accordance with relevant guidelines and regulations. No identifiers were collected during the study. In compliance with ethical research practices, informed consent was obtained from all participants before initiating the survey. Attached to the survey was a comprehensive cover letter outlining the purpose of the study, the procedure involved, the approximate time to complete the survey, and assurances of anonymity and confidentiality. It also emphasized that participation was completely voluntary, and participants could withdraw at any time without any consequences. The cover letter also included contact information of the researchers for any questions or concerns the participants might have regarding the study. Participants were asked to read through this information carefully and were instructed to proceed with the survey only if they understood and agreed to the terms described, effectively providing their consent to participate in the study.

### Study design and setting

This research adopted a cross-sectional survey design and convenience sampling strategy to gain insights into the experiences and perspectives of patients undergoing treatment or having completed their treatment for gastrointestinal cancer. A web-based survey was hosted on Qualtrics and disseminated by Centiment, an audience paneling service^38^, facilitating a broad reach to potential participants across different geographic locations in the United States. The survey distribution channels included various online platforms such as social media, cancer forums, and other related websites. Data collection occurred throughout March 2023.

### Participants

Participants eligible for the study were individuals who were undergoing treatment or had completed their treatment for gastrointestinal cancer in the United States. There were no further specified exclusion criteria; however, the overarching requirement was the participant's connection to the specified medical condition.

Potential participants encountered the survey through its dissemination on online platforms, including social media channels and specialized cancer forums. Given the voluntary nature of online surveys, participants self-selected into the study by choosing to engage and complete the survey.

### Variables and data sources

The study encompassed 20 observed variables as shown in Table 1. Primary outcomes of interest included patients' suicidal ideation and mental health (depression) status. The question regarding suicidal ideation were adapted from the Columbia-Suicide Severity Rating Scale (C-SSRS)^39^. The responses were recorded as “only one time,” “a few times,” “a lot,” “all the time,” and “I don't know.” Mental health (depression) related questions were adapted from the Patient Health Questionnaire (PHQ-4)^40,41^. Respondents were recorded as “all the time,” “most of the time,” “rarely,” and “never”. Increasing value of mental health would therefore signify lower depression.

**Table 1:** The survey instrument.

**Questions**	
Suicidal ideation Q1. Have you thought about being dead or what it would be like to be dead? (SI 1) Q2. Have you wished you were dead or wished you could go to sleep and never wake up? (SI 2) Q3. Do you wish you were not alive anymore? (SI 3) Q4. Have you thought about doing something to make yourself not alive anymore? (SI 4) Q5. Have you had any thoughts about killing yourself? (SI 5) Q6. Have you thought about how you would make yourself not alive anymore (kill yourself)? (SI 6) Q7. When you thought about making yourself not alive anymore (or killing yourself), did you think that this was something you might do? (SI 7) Q8. Have you decided how or when you would make yourself not alive anymore/kill yourself? (SI 8)	
Mental health (Inverse measure of depression) Q9. During your cancer treatment, did you ever experience: Loneliness (MH 1) Q10. During your cancer treatment, did you ever experience: Hopelessness (MH 2) Q11. During your cancer treatment, did you ever experience: Nervousness (MH 3) Q12. During your cancer treatment, did you ever experience: Sever Sadness (MH 4)	
Family burden Q13. Have you ever felt like your family was carrying a heavy burden because of your treatment?	
Internet information utilization Q14. Have you ever used the internet to learn about your cancer and treatment plan	
Emotional support Q15. During cancer treatment, did you ever share your thoughts and emotions with someone?	
Age group Q16. What is your age (years)?	
Gender Q17. With which of the following gender do you identify yourself as	
Annual Income Q18. What is your annual household income	
Education Q19. What is the highest grade or level of schooling you completed?	
Household occupants Q20. Including yourself, how many people live in your household?	

Variables hypothesized to influence or predict the outcomes included family burden, internet information utilization, and emotional support. These were measured using a five-point Likert scale, ranging from "never" to "always." Demographic variables such as age, gender, income, education, and household occupants were considered as potential confounders, which could influence the relationship between the predictors and the outcomes.

The study explored several variables as potential effect modifiers, specifically investigating their moderation effects on the relationship between mental health (depression) and suicidal ideation in patients with gastrointestinal cancer. These include education, gender, income, household occupants, emotional support, internet information utilization, age, and family burden.

All participants, irrespective of their source of recruitment (e.g., social media or cancer forums), were exposed to the same assessment methods (survey instrument).

### Quantitative variables

The study employed ordinal variables, including responses captured using Likert scales. These types of variables represent ordered categories, allowing for a rank order of responses but without assuming consistent intervals between categories. Ordinal variables were primarily analyzed using non-parametric statistical methods suitable for ordinal data. Likert scales captured data in ordered categories. The analysis did not assume equidistant intervals between the response categories.

Groupings inherent to the Likert scales and other ordinal variables were chosen based on their relevance to the research questions and to provide meaningful distinctions in the participants' responses. The aim was to capture nuanced perspectives and experiences of gastrointestinal cancer patients, ensuring the scales and categories aligned with the context and depth of the study's objectives.

### Bias

Potential biases inherent to this study include sampling, response, measurement/instrument, non-response, selection, and recall biases. The targeted audience of patients undergoing or having completed treatment for gastrointestinal cancer, combined with the use of an online survey and Centiment's audience paneling service, may not provide a fully generalizable picture to all cancer patients or the general population. While the anonymity of responses and inclusion of checking questions aimed to reduce response biases, the adaptation of instruments like the C-SSRS and PHQ-4, coupled with the self-reported nature of the data, might introduce measurement, and recall biases. Despite these considerations, the study utilized established instruments and took measures to ensure participant understanding and candor. Readers should interpret findings in light of these potential biases and the inherent limitations of online survey research.

### Study size

The sample size for this study was not pre-determined through formal statistical power calculations. Instead, it was driven by a convenience sampling approach. We disseminated the survey on various online platforms, including social media, cancer forums, and other related websites, open to any eligible individual willing to participate. Given the nature of online dissemination and voluntary participation, we had limited control over the final sample size. The received responses are thus representative of those who encountered the survey and chose to engage, rather than a statistically calculated representation of the gastrointestinal cancer patient population.

### Statistical methods

All the analyses were performed using the SEMinR package^42^ in R^43^. First the descriptive statistics of all the survey questions were calculated. Then question SI 1 through SI 8 and MH 1 through MH 4 were combined to form the latent constructs–Suicidal ideation and Mental Health (depression), respectively. The convergent validity of these two latent constructs were measured using: (a) Cronbach’s alpha requiring to be greater than 0.70; (b) outer loadings greater than 0.50^44^. Discriminant validity was assessed using the Fornell-Larcker criterion^45^ and the Heterotrait-monotrait (HTMT) ratio requiring to be less than 0.85^46^. We also examined multicollinearity using the variance inflation factor (VIF) to be less than 2.5^47^.

Upon validating the laten constructs, we calculated a total score (summation) representing suicidal ideation and mental health. The calculated score was used for preliminary bivariate and correlation analysis.

We used the non-parametric Partial Least Squares-Structural Equation Modeling (PLS-SEM)^48^ approach to examine our hypotheses. We defined an estimated model (structural model) and controlled for age, gender, education, annual income, and household occupants. The model fit was then evaluated by R-squared and adjusted R-squared values^49^. We measure the significance of the model by bootstrapping method with 10,000 subsamples^50^, which involves resampling with replacement from the original sample to generate a new sample and subsequently estimating the model on the new sample. This process was repeated multiple times to produce a distribution of estimates, enabling more precise population inferences. We tested significance at a 95% confidence level (two-tailed).

Additionally, we performed moderation and multigroup analysis to investigate if the observed relationship between the independent and dependent variable changes depending on the levels of a third variable, known as the moderator variable^51^ or differ for patients undergoing GIC treatment and those who have completed their treatment, respectively.

## Results

### Participants and data description

**Figure 2:**
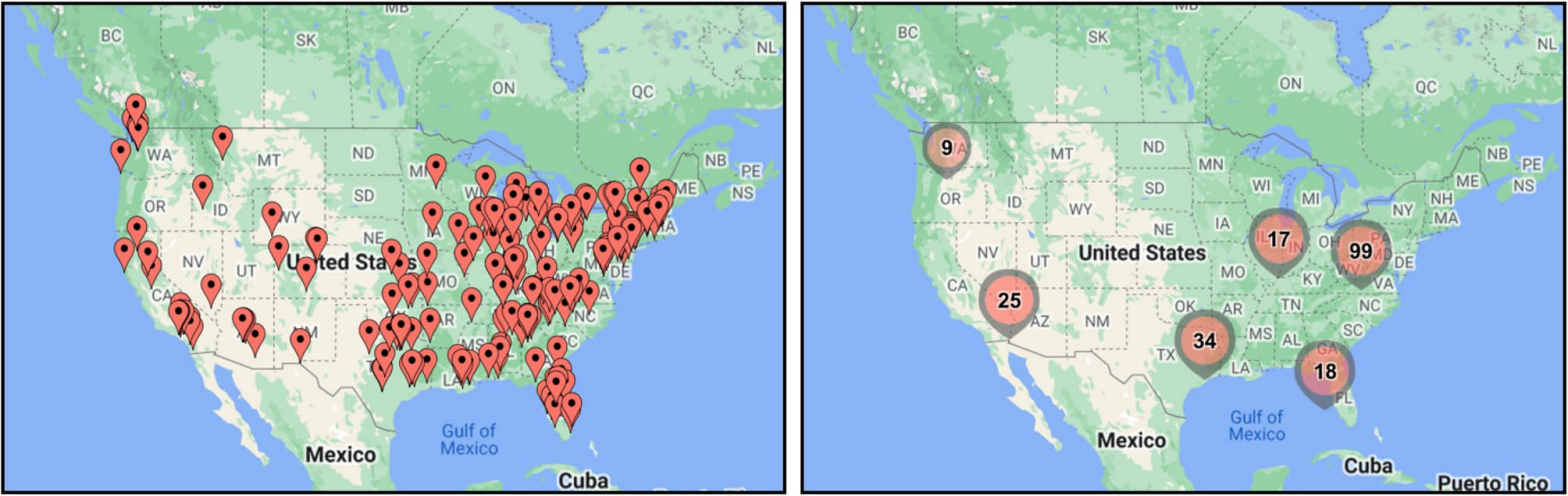
Approximate location of study participants based on longitude and latitude data.

In this study, 202 respondents completed the survey of which 78 were undergoing treatment related to their GIC diagnosis. Figure 2 displays the geographical distribution of study participants across the United States, visualized using two distinct map representation. The left map uses individual red pin markers to indicate the approximate location of each study participant. A dense clustering of these pins can be observed primarily in the eastern half of the US, indicating a high concentration of participants in this region. The western half has a sparser distribution, with the exception of some clusters on the west coast. The right map with aggregated circular markers represents the number of participants in specific regions. The size of the circle and the number within it denote the count of participants. For example, the circle near Kentucky indicates a high concentration of 99 participants in that vicinity. Similarly, the map showcases other significant concentrations in areas such as Arizona (25 participants), Missouri (34 participants), Michigan (17 participants), and Florida (18 participants).

Table 2 shows the data description of all the variables used in the study. Of all the respondents, 110 identified themselves as females. Our participant cohort comprised patients with various types of GIC of which 26 respondents reported having multiple GICs (comorbidities). Remaining 176 respondents reported to have single GIC diagnosis (113 colorectal cancer, 12 esophageal cancer, 12 stomach cancer, 7 pancreatic cancer, 7 liver cancer, 6 anal cancer, 6 small intestine cancer, and 13 participants specified other types of gastrointestinal cancers that were not explicitly listed in the survey options.

**Table 2:** Descriptive statistics of study variables (n = 202).

		Are you currently undergoing the cancer treatment?		*P value*
		No (n = 124)	Yes (n = 78)	< *.001*
		N (%)		
S1	Only once	10 (8.10)	6 (7.70)	
	A few times	43 (34.70)	31 (39.70)	
	A lot	9 (7.30)	11 (14.10)	
	All the time	6 (4.80)	10 (12.80)	*.041*
	I don’t know	56 (45.20)	20 (25.60)	*.005*
S2	Only once	9 (7.30)	10 (12.80)	
	A few times	27 (21.80)	25 (32.10)	
	A lot	7 (5.60)	10 (12.80)	
	All the time	7 (5.60)	2 (2.60)	
	I don’t know	74 (59.70)	31 (39.70)	*.006*
S3	Only once	9 (7.30)	12 (15.40)	
	A few times	12 (9.70)	19 (24.40)	*.005*
	A lot	4 (3.20)	9 (11.50)	*.019*
	All the time	8 (6.50)	3 (3.80)	
	I don’t know	91 (73.40)	35 (44.90)	< *.001*
S4	Only once	13 (10.50)	14 (17.90)	
	A few times	9 (7.30)	17 (21.80)	*.003*
	A lot	3 (2.40)	7 (9.00)	*.037*
	All the time	7 (5.60)	5 (6.40)	
	I don’t know	92 (74.20)	35 (44.90)	*.000*
S5	Only once	11 (8.90)	11 (14.10)	
	A few times	16 (12.90)	18 (23.10)	
	A lot	1 (< 1)	7 (9.00)	*.004*
	All the time	7 (5.60)	6 (7.70)	
	I don’t know	89 (71.80)	36 (46.20)	< *.001*
S6	Only once	13 (10.50)	11 (14.10)	
	A few times	13 (10.50)	20 (25.60)	*.005*
	A lot	2 (1.60)	6 (7.70)	*.031*
	All the time	7 (5.60)	6 (7.70)	
	I don’t know	89 (71.80)	35 (44.90)	< *.001*
S7	Only once	10 (8.10)	15 (19.20)	*.019*
	A few times	8 (6.50)	12 (15.40)	*.038*
	A lot	4 (3.20)	7 (9.00)	
	All the time	7 (5.60)	6 (7.70)	
	I don’t know	95 (76.60)	38 (48.70)	< *.001*
S8	Only once	11 (8.90)	12 (15.40)	
	A few times	7 (5.60)	15 (19.20)	*.003*
	A lot	3 (2.40)	5 (6.40)	
	All the time	8 (6.50)	4 (5.10)	
	I don’t know	95 (76.60)	42 (53.80)	*.001*
MH1	All the time	18 (14.50)	12 (15.40)	
	Most of the time	31 (25.00)	24 (30.8)	
	Rarely	42 (33.90)	30 (38.50)	
	Never	33 (26.60)	12 (15.40)	
MH2	All the time	7 (5.60)	10 (12.80)	
	Most of the time	33 (26.60)	30 (38.50)	
	Rarely	46 (37.10)	23 (29.50)	
	Never	38 (30.60)	15 (19.20)	
MH3	All the time	20 (16.10)	25 (32.10)	*.008*
	Most of the time	51 (41.10)	31 (39.70)	
	Rarely	38 (30.60)	18 (23.10)	
	Never	15 (12.10)	4 (5.1)	
MH4	All the time	9 (7.30)	15 (19.20)	*.010*
	Most of the time	32 (25.80)	27 (34.60)	
	Rarely	41 (33.10)	19 (24.40)	
	Never	42 (33.90)	17 (21.80)	
Family burden	Never	32 (25.80)	12 (15.40)	
	Sometimes	47 (37.90)	21 (26.90)	
	About half the time	12 (9.70)	19 (24.40)	*.005*
	Most of the time	19 (15.30)	15 (19.20)	
	Always	14 (11.30)	11 (14.10)	
Internet information utilization	Never	15 (12.10)	4 (5.10)	
	Sometimes	45 (36.30)	21 (26.90)	
	About half the time	17 (13.70)	11 (14.10)	
	Most of the time	26 (21.00)	25 (32.10)	
	Always	21 (16.90)	17 (21.80)	
Emotional support	Never	16 (12.90)	5 (6.40)	
	Sometimes	49 (39.50)	23 (29.50)	
	About half the time	17 (13.70)	13 (16.70)	
	Most of the time	20 (16.10)	25 (32.10)	*.008*
	Always	22 (17.70)	12 (15.40)	
Age group	18 to 24	1 (< 1.00)	0 (0)	
	25 to 35	1 (< 1.00)	0 (0)	
	36 to 45	8 (6.50)	25 (32.10)	< *.001*
	46 to 55	22 (17.70)	19 (24.40)	
	56 to 65	34 (27.40)	20 (25.60)	
	More than 65	58 (46.80)	14 (17.90)	< *.001*
Gender	Male	51 (41.10)	41 (52.60)	
	Female	73 (58.90)	37 (47.40)	
	Non-binary/third gender	0 (0)	0 (0)	
	Prefer not to say	0 (0)	0 (0)	
Annual income	Less than $20,000	20 (16.10)	3 (3.80)	*.007*
	$20,000 to < $35,000	27 (21.80)	17 (21.80)	
	$35,000 to < $50,000	22 (17.70)	11 (14.10)	
	$50,000 to < $80,000	20 (16.10)	22 (28.20)	*.039*
	$80,000 to < $100,000	8 (6.60)	8 (10.30)	
	$100,000 to < $120,000	9 (7.30)	5 (6.40)	
	$120,000 to < $150,000	5 (4.00)	4 (5.10)	
	$150,000 to < $200,000	10 (8.10)	4 (5.10)	
	More than $200,000	3 (2.40)	4 (5.10)	
Education	Less than high school	0 (0)	1 (1.30)	
	High school graduate	16 (12.90)	14 (17.90)	
	Some college	34 (27.40)	20 (25.60)	
	2-year degree	29 (23.40)	7 (9.00)	*.009*
	4 year degree	25 (20.20)	25 (32.10)	
	Professional degree	16 (12.90)	9 (11.50)	
	Doctorate	4 (3.20)	2 (2.60)	
Household occupants	1	28 (22.60)	12 (15.40)	
	2	65 (52.40)	22 (28.20)	*.001*
	3	17 (13.70)	18 (23.10)	
	4	9 (7.30)	14 (17.90)	*.020*
	5	3 (2.40)	4 (5.10)	
	6	2 (1.60)	7 (9.00)	*.014*
	More than 6	0 (0)	1 (1.30)	

Significant values are in italic.

**Figure 3:**
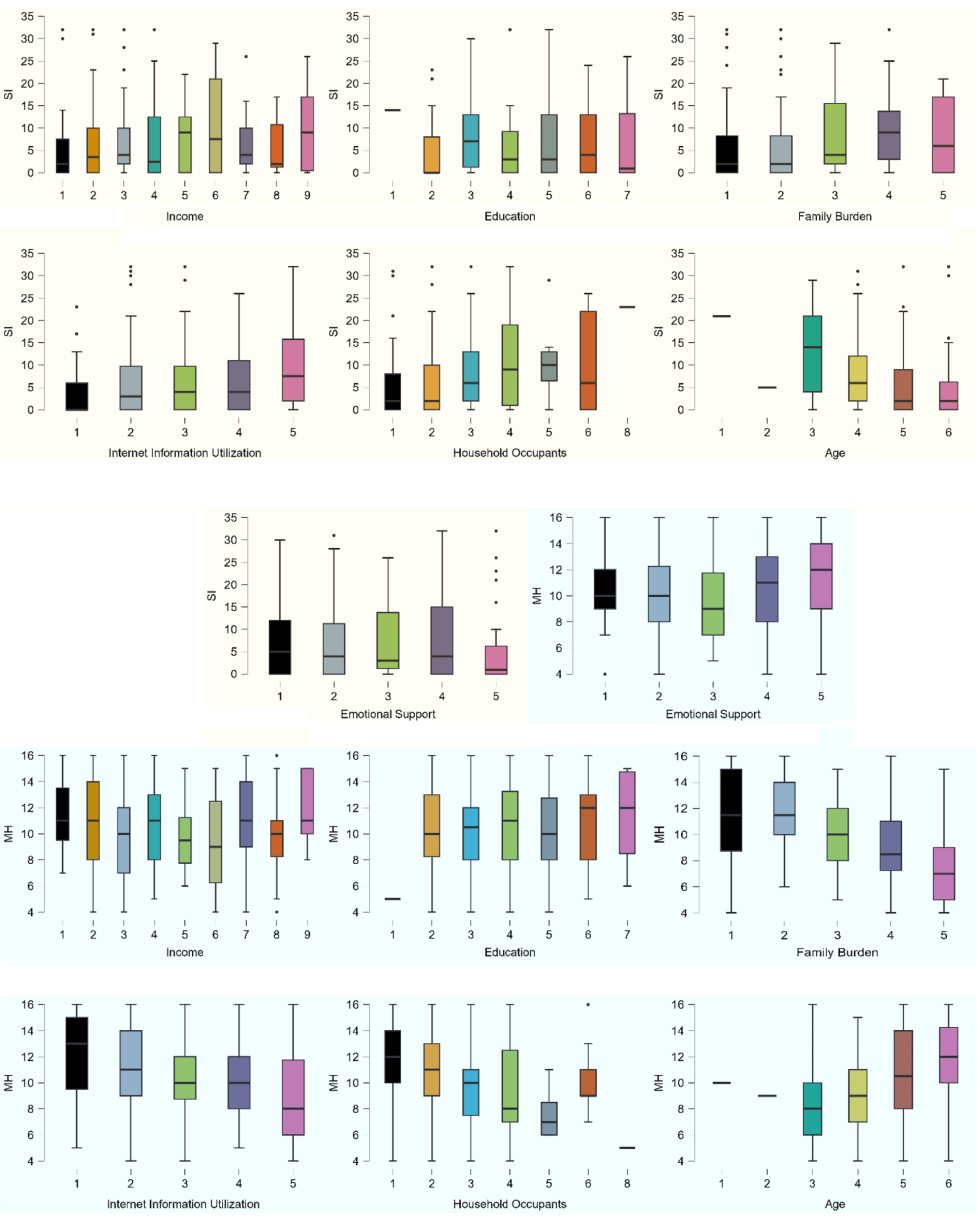
Distribution of Suicidal Ideation (SI) and Mental Health (MH) Across Socio-Demographic and Environmental Factors. Box plots represent the spread and central tendency of SI and MH scores across various sociodemographic categories, including Income, Education, Family Burden, Internet Information Utilization, Household Occupants, Age, and Emotional Support.

Figure 3 is organized into box plots illustrating the distribution of suicidal ideation (SI) and mental health (MH, an inverse measure of depression) in relation to various sociodemographic and environmental factors, including Income, Education, Family Burden, Internet Information Utilization, Household Occupants, Age, and Emotional Support. For SI, outlier data points are prevalent across almost all categories, suggesting that while there are general patterns, individual experiences can vary significantly. MH scores seem to be more consistent across categories, but certain groups, especially those with lower levels of emotional support or those at specific age ranges, tend to deviate from the norm.

**Figure 4:**
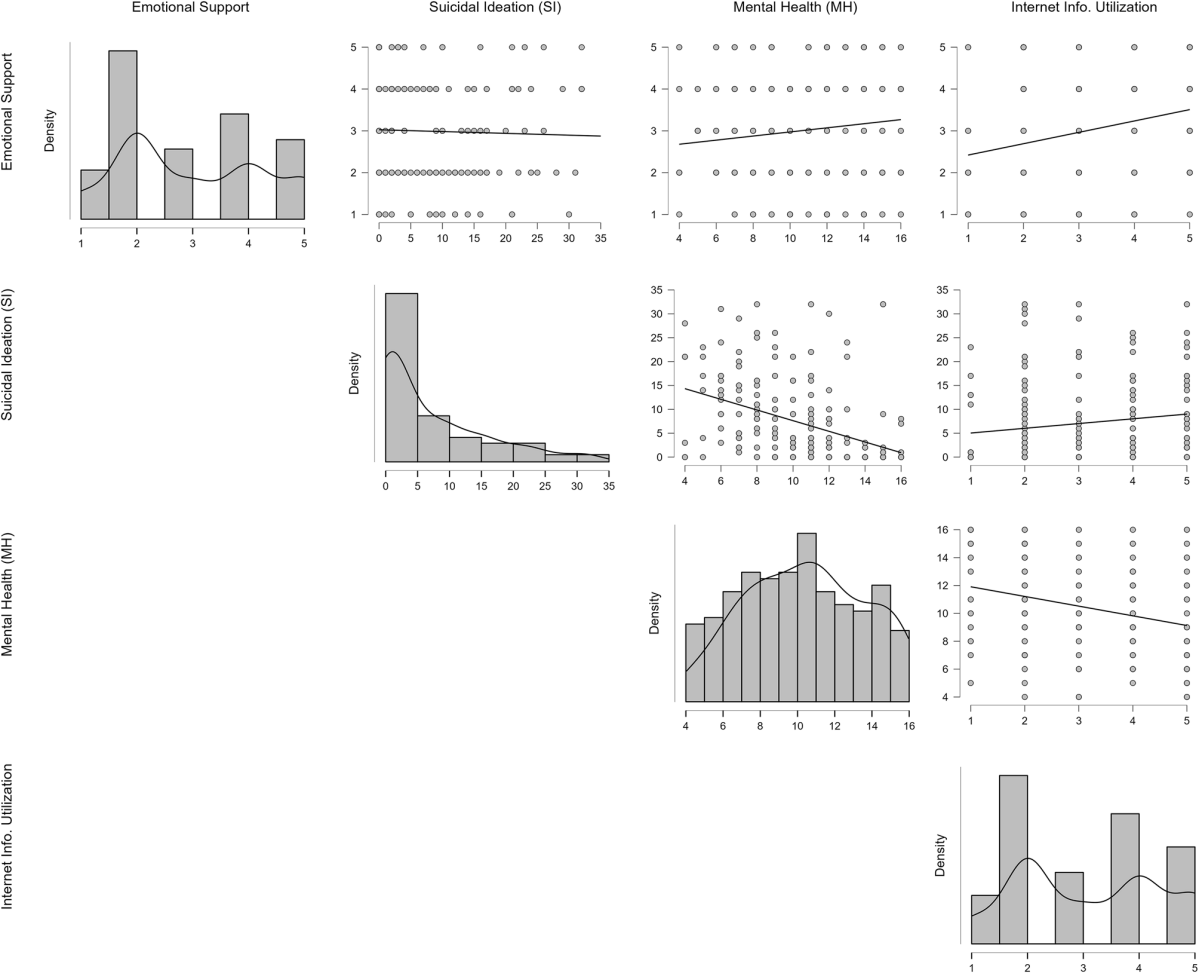
Correlations of Suicidal Ideation (SI) and Mental Health (MH) in Context of Emotional Support and Internet Information Utilization: The figure integrates histograms with density plots to show the distributions of Emotional Support, SI, MH, and Internet Information Utilization. Adjacent scatter plots illustrate the bivariate relationships between these variables. Trend lines provide a visual guide to the overall direction of these relationships.

Figure 4 shows the correlation between emotional burden, family support, internet information utilization, suicidal ideation, and mental health. The figure juxtaposes the distributions of Suicidal Ideation (SI), Mental Health (MH), Emotional Support, and Internet Information Utilization using histograms integrated with density plots. Notably, while SI's distribution is right-skewed, indicating most individuals have low SI, MH exhibits a slight left skew, with a majority having moderate scores. There's a distinct inverse relationship between SI and MH, implying higher suicidal ideation corresponds with poorer mental health. The role of Emotional Support and Internet Information Utilization is more nuanced; where a higher emotional support level appears to result in a tighter spread of both SI and MH scores and the degree of Internet Information Utilization present a clear influence on MH.

**Figure 5:**
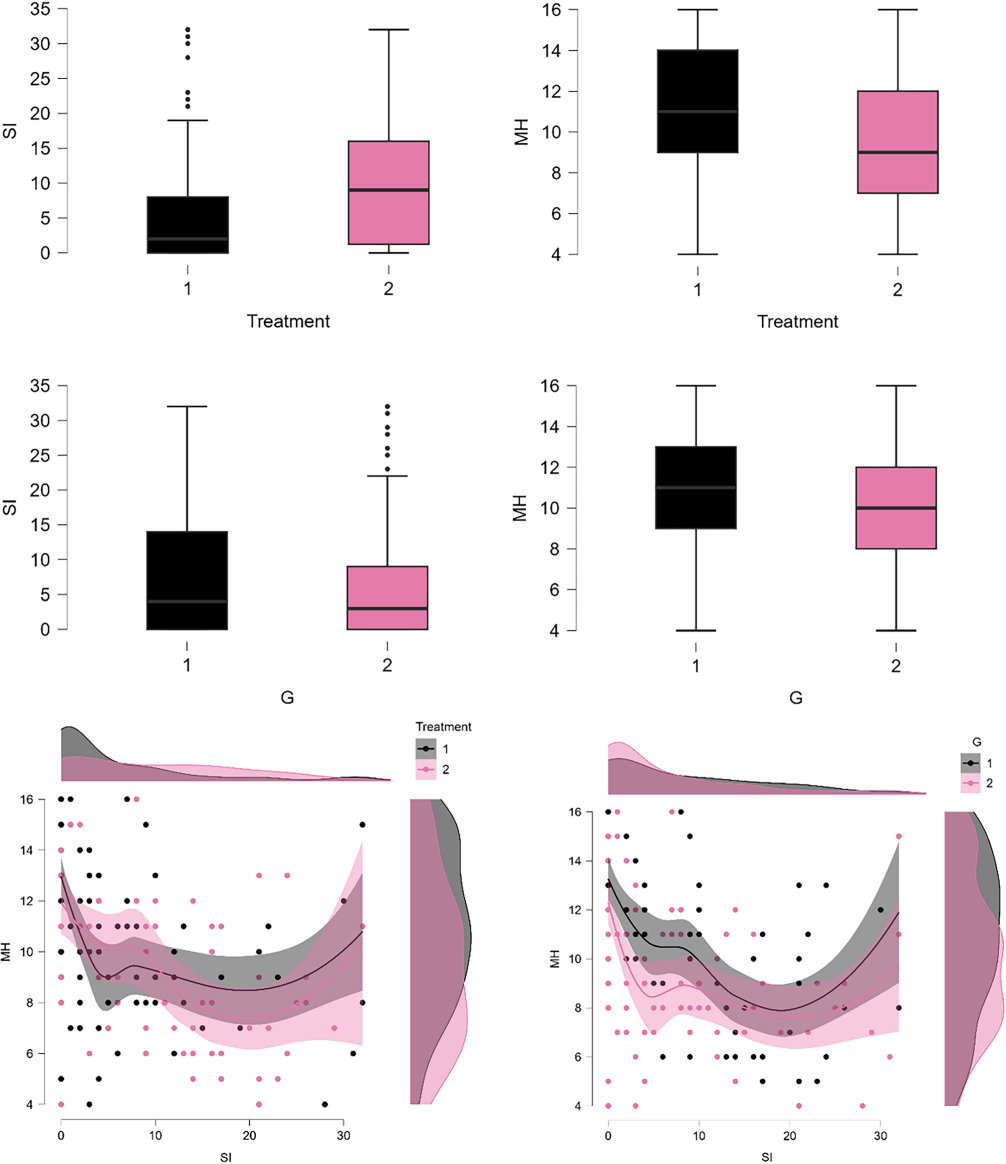
Comparative Analysis of Suicidal Ideation (SI) and Mental Health (MH) Scores Across Treatment and Gender Groups. The top panel presents box plots illustrating the distribution of suicidal ideation (SI) and mental health (MH, an inverse measure of depression) scores for two treatment groups: patients who completed GIC treatment (treatment 1) and those undergoing GIC treatment (treatment 2). The middle panel shows the distribution of suicidal ideation (SI) and mental health (MH, an inverse measure of depression) scores for two gender groups: (males-G1; females-G2). The bottom panel depicts scatter plots with density shading, highlighting the relationship between SI and MH scores, stratified by treatment and gender groups.

Figure 5 shows a series of box plots (top and middle panel) and scatter plots (bottom panel), representing suicidal ideation (SI) and mental health (MH), grouped based on treatment status (treatment 1: completed GIC treatment; treatment 2: undergoing GIC treatment) and gender (G1: male; G2: female). Cancer patients who completed the GIC treatment exhibited a specific distribution of SI, as shown in top left box plot for Treatment 1 (Fig. 5). The median SI was noticeably lower in this group compared to those undergoing the treatment. Some data points, lying beyond the upper quartile, suggest the presence of outliers or individuals with exceptionally high SI even after completing the treatment. Patients currently in the GIC treatment phase as shown in the top left box plot for Treatment 2, showcased a higher median SI, accompanied by a broader range of SI scores. When stratified by gender, as shown in the left boxplot of the middle panel, males (G1) demonstrated a wider distribution of SI values with a comparable median compared to females (G2). A few outliers were also observed above the upper quartile for the female group.

As shown in the top right box plot (Fig. 5), the distribution of mental health (MH, an inverse measure of depression), for patients who completed the GIC treatment (treatment 1) appeared to have a median slightly higher, indicating less depression or better mental health, compared to those still undergoing treatment (treatment 2). According to the middle panel right box plot the spread of mental health (MH) values for both genders appears to be almost identical, indicating similar central tendencies for mental health levels.

For patients who completed their GIC treatment (treatment 1), the bottom left scatter plot (Fig. 5) showcases a prominent U-shaped relationship between SI and MH. As SI values rise, MH scores first dip, indicating heightened depression, before ascending, signifying reduced depression. The higher density region, shown by the dark shade of the U-curve, suggests that most patients in this group fall within this range. Patients undergoing GIC treatment (treatment 2) also demonstrate a U-shaped trend between SI and MH, but the curvature is more gradual than Treatment 1. Notably, there's a higher concentration of patients with low SI and high MH scores, suggesting less suicidal ideation and depression for a significant portion of this group. The shaded regions surrounding each regression line represent 95% confidence intervals. The overlap in these intervals, especially in the mid-range of SI values, suggests similarities in SI-MH relationships across the two treatments within this range. When stratified by gender, as shown in bottom right scatter plot (Fig. 5), the male group showcases a pronounced U-shaped curve between SI and MH. There's a high-density region around the mid-range of SI values, indicating that a significant portion of male patients falls within this SI-MH range. The female group also exhibits a U-shaped relationship between SI and MH. The curve's depression seems less pronounced, with the lowest point of MH (highest depression) being higher than that of the male group. The density indicates that many female patients have low SI values and high MH scores, pointing to reduced suicidal ideation and depression for a notable portion of this group.

### Measurement model’s reliability and validity

The measurement model demonstrated strong internal consistency and reliability for the Suicidal Ideation construct. Both Cronbach’s alpha and composite reliability surpassed the 0.70 benchmark, registering values of 0.862 and 0.906, respectively. With an Average Variance Extracted (AVE) value of 0.708, which exceeds the 0.5 threshold, the convergent validity of the construct is also established. The Mental Health (depression) construct showcased strong reliability, with Cronbach’s alpha of 0.954 and a composite reliability of 0.962. Its AVE value, 0.759, affirmed its convergent validity.

Discriminant validity for the Suicidal Ideation construct was evidenced by the square root of its AVE (0.871) being greater than its correlation with Mental Health (0.841). Furthermore, HTMT values under 0.85 support the discriminant validity of both constructs. Variance Inflation Factor (VIF) values for Suicidal Ideation ranged from 1.050 to 1.595, and for Mental Health, they were between 1.027 and 1.395. These values indicate that multicollinearity is not a significant concern in the model.

### Structural model

**Figure 6:**
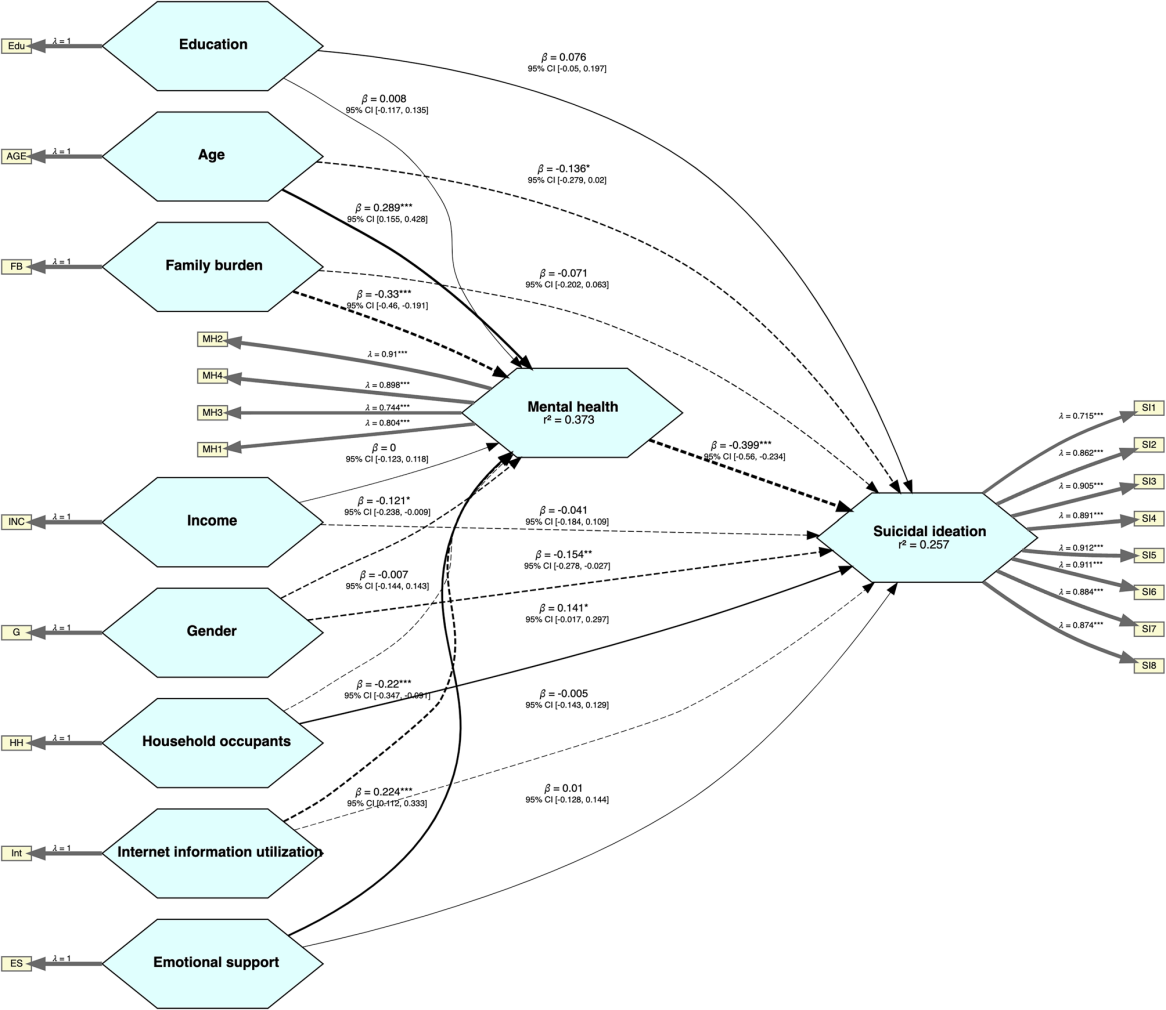
The structural model illustrates the relationships between suicidal ideation, mental health (depression), family burden, internet information utilization, emotional support, age, gender, income, education, and household occupants.

Figure 6 illustrates the structural model, where (λ) indicates the factor loading of the different latent constructs and (β) indicates the standardized coefficient/estimate. The solid lines in the figure represent positive relationships whereas the dotted (dashed) lines represent inverse relationships.

The model exhibited an R-squared value of 0.373 and 0.257 for mental health and suicidal ideation, respectively. Table 3 shows the direct relationships between the study variables. The analysis indicated that the mental health (depression) of GIC patients was significantly inversely correlated with the incidence of suicidal ideation (β = −0.399), thus supporting hypothesis H1. The analysis also showed a significant inverse correlation between family burden and mental health (depression) (β = −0.330), confirming hypothesis H2. However, the relationship between family burden and suicidal ideation was not significant (β = −0.071), leading to the rejection of hypothesis H3. Internet usage for health-related information significantly inversely correlated with mental health (depression) (β = −0.220), substantiating hypothesis H4, but was not significantly related to suicidal ideation (β = −0.005), causing hypothesis H5 to be rejected. Emotional support positively correlated with mental health (depression) (β = 0.224), supporting hypothesis H6, but was not significantly related to suicidal ideation (β = 0.010), rejecting hypothesis H7.

**Table 3:** Direct influence of family burden, internet information utilization, and emotional support on mental health and suicidal ideation, with control variables including education, gender, income, household occupants, and age.

Null hypotheses	Path	β	SD	T Stat	CI [5%, 95%]
H1: Fail to reject	Mental health → Suicidal ideation	− 0.399	0.082	− 4.847	[− 0.560, − 0.234] *
H2: Fail to reject	Family burden → Mental health	− 0.330	0.068	− 4.838	[− 0.460, − 0.191] *
H3: Reject	Family burden → Suicidal ideation	− 0.071	0.067	− 1.059	[− 0.202, 0.063]
H4: Fail to reject	Internet information utilization → Mental health	− 0.220	0.065	− 3.381	[− 0.347, − 0.091] *
H5: Reject	Internet information utilization → Suicidal Ideation	− 0.005	0.069	− 0.076	[− 0.143, 0.129]
H6: Fail to reject	Emotional support → Mental health	0.224	0.056	3.975	[0.112, 0.333] *
H7: Reject	Emotional support → Suicidal ideation	0.010	0.069	0.140	[− 0.128, 0.144]
Control variables					
Education → Mental health		0.008	0.064	0.126	[− 0.117, 0.135]
Education → Suicidal ideation		0.076	0.063	1.203	[− 0.050, 0.197]
Gender → Mental health		− 0.121	0.058	− 2.071	[− 0.238, − 0.009] *
Gender → Suicidal ideation		− 0.154	0.065	− 2.386	[− 0.278, − 0.027] *
Income → Mental health		0.000	0.062	0.005	[− 0.123, 0.118]
Income → Suicidal ideation		− 0.041	0.075	− 0.538	[− 0.184, 0.109]
Household occupants → Mental health		− 0.007	0.074	− 0.090	[− 0.144, 0.143]
Household occupants → Suicidal ideation		0.141	0.080	1.752	[− 0.017, 0.297]
Age → Mental health		0.289	0.069	4.195	[0.155, 0.428] *
Age → Suicidal ideation		− 0.136	0.077	− 1.763	[− 0.279, 0.020]

The asterisks (*) in the table denote significant relationships.

β = Standardized path coefficient.

SD = Standard deviation.

Regarding control variables, age showed a significant positive relationship with mental health (depression) (β = 0.289) and a negative but insignificant relationship with suicidal ideation (β = − 0.136). Gender significantly negatively correlated with both mental health (depression) (β = −0.121) and suicidal ideation (β = − 0.154). Other control variables such as education, income, and the number of household occupants did not have a significant relationship with either mental health (depression) or suicidal ideation.

Table 4 highlights the critical mediating role of mental health (depression) in various relationships impacting suicidal ideation in GIC patients. Notably, significant indirect effects were observed in the path from family burden (β = 0.132), internet information utilization (β = 0.088), emotional support (β = − 0.089), gender (β = 0.048), and age (β = − 0.116) to suicidal ideation, mediated through mental health (depression). These effects underscore the indirect influence these factors exert on suicidal ideation by shaping mental health (depression outcomes). Conversely, education, income, and household occupants did not demonstrate significant indirect effects on suicidal ideation via depression.

**Table 4:** Indirect influence of family burden, internet information utilization, and emotional support on mental health and suicidal ideation, with control variables including education, gender, income, household occupants, and age.

Paths	β	SD	T Stat	CI [5%, 95%]
Family burden → Mental health → Suicidal Ideation	0.132	0.038	3.482	[0.064, 0.212] *
Internet information utilization → Mental health → Suicidal Ideation	0.088	0.032	2.788	[0.033, 0.155] *
Emotional support → Mental health → Suicidal Ideation	− 0.089	0.030	− 2.981	[− 0.154, − 0.037] *
Education → Mental health → Suicidal Ideation	− 0.003	0.027	− 0.122	[− 0.058, 0.048]
Gender → Mental health → Suicidal Ideation	0.048	0.026	1.848	[0.003, 0.105] *
Income → Mental health → Suicidal Ideation	− 0.000	0.025	− 0.005	[− 0.049, 0.051]
Household occupants → Mental health → Suicidal Ideation	0.003	0.030	0.088	[− 0.060, 0.062]
Age → Mental health → Suicidal Ideation	− 0.116	0.039	− 2.984	[− 0.199, − 0.050] *

The asterisks (*) in the table denote significant relationships

β = Standardized path coefficient

SD = Standard deviation

### Moderation effect

**Figure 7:**
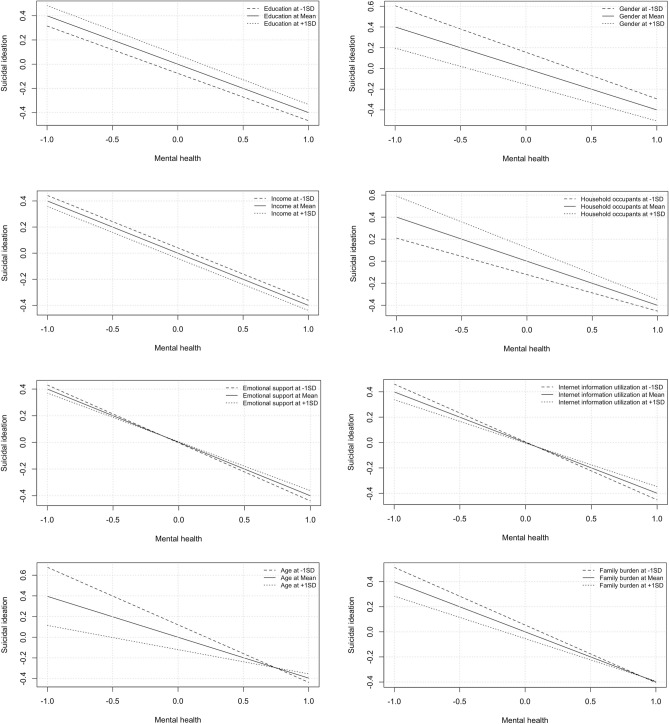
Moderation effects of education, gender, income, household occupants, emotional support, internet information utilization, age, and family burden on the relationship between mental health (depression) and suicidal ideation in patients with gastrointestinal cancer.

As shown in Table 5, the interaction between mental health (depression) and age (β = 0.161) significantly moderated the relationship with suicidal ideation. This implies that the influence of mental health (depression) on suicidal ideation in GIC patients differs depending on the age of the patient, with older patients showing a more significant effect. Conversely, no significant moderating effects were observed between mental health (depression) and education, gender, income, household occupants, internet information utilization, emotional support, or family burden on suicidal ideation. Thus, the influence of mental health (depression) on suicidal ideation remained relatively constant across these categories (education, gender, income, household occupants, internet usage, emotional support, and family burden), suggesting that these factors do not significantly alter the relationship between mental health (depression) and suicidal ideation. This underscores the unique challenges faced by older patients and the importance of age-specific considerations in interventions addressing mental health (depression) and suicidal ideation in this population. Figure 7 illustrates all the moderation effects assessed in the study.

**Table 5:** Moderating effects of education, gender, income, household occupants, age, internet information utilization, emotional support, and family burden on the relationship between mental health and suicidal ideation.

Paths	β	SD	T Stat	CI [5%, 95%]
Mental health*Education → Suicidal ideation	− 0.009	0.049	− 0.176	[− 0.101, 0.086]
Mental health* Gender → Suicidal ideation	0.049	0.067	0.734	[− 0.079, 0.182]
Mental health* Income → Suicidal ideation	0.002	0.061	0.027	[− 0.120, 0.121]
Mental health* Household occupants → Suicidal ideation	− 0.069	0.067	− 1.032	[− 0.206, 0.061]
Mental health* Age → Suicidal ideation	0.161	0.084	1.921	[0.005, 0.334] *
Mental health* Internet information utilization → Suicidal ideation	0.057	0.062	0.922	[− 0.067, 0.176]
Mental health* Emotional support → Suicidal ideation	0.034	0.073	0.473	[− 0.114, 0.169]
Mental health* Family burden → Suicidal ideation	0.060	0.062	0.979	[− 0.064, 0.117]

The asterisks (*) in the table denote significant relationships.

β = Standardized path coefficient.

SD = Standard deviation.

### Multigroup analysis

Table 6 shows the comparison of observed relationships across two groups. Group 1 corresponds to participants who completed their GIC treatment and Group 2 corresponds to those who were undergoing their GIC treatment during the time of this study. We noted a significant difference in the influence of emotional support on mental health (depression) across the two groups, where the impact was significantly higher group 1. No other significant differences were observed.

**Table 6:** Multigroup analysis comparing the direct influence of family burden, internet information utilization, and emotional support on mental health and suicidal ideation, for patients undergoing gastrointestinal cancer treatment and survivors.

Path	Group 1 (β)	Group 2 (β)	*P-Value*
Mental health → Suicidal ideation	− 0.415	− 0.377	*0.575*
Family burden → Mental health	− 0.283	− 0.441	*0.139*
Family burden → Suicidal ideation	− 0.096	− 0.064	*0.594*
Internet information utilization → Mental health	− 0.21	− 0.154	*0.681*
Internet information utilization → Suicidal ideation	0.024	− 0.047	*0.310*
Emotional support → Mental health	0.363	0.038	0.002*
Emotional support → Suicidal ideation	− 0.002	0.006	*0.525*
Control variables			
Education → Mental health	− 0.050	0.095	*0.873*
Education → Suicidal ideation	− 0.039	0.176	*0.950*
Gender → Mental health	− 0.210	− 0.084	*0.836*
Gender → Suicidal ideation	− 0.152	− 0.136	*0.540*
Income → Mental health	0.023	0.030	*0.507*
Income → Suicidal ideation	− 0.062	0.0124	*0.706*
Household occupants → Mental health	− 0.049	0.085	*0.825*
Household occupants → Suicidal ideation	0.095	0.091	*0.463*
Age → Mental health	0.186	0.376	*0.916*
Age → Suicidal ideation	0.002	− 0.231	*0.076*

β = Standardized path coefficient.

Group 1 = participants who completed their gastrointestinal cancer treatment.

Group 2 = participants undergoing gastrointestinal cancer treatment.

The asterisks (*) in the table denote the significance.

Significant values are in italic.

## Discussion

Our research elucidates the direct and indirect associations of family burden, utilization of internet-based health information, and emotional support with mental health (depression) and suicidal ideation among patients diagnosed with gastrointestinal cancer. This comprehensive assessment also considers various moderating factors such as education, gender, income, household occupants, and age. The implications of these findings hold significant value for health practitioners and policymakers in formulating effective strategies for mental health (depression) improvement and suicide prevention among this patient group.

The findings from the present study hold interesting parallels and contribute valuable insights to the existing body of research on the psychological well-being of cancer patients. Consistent with existing evidence, our research substantiated the presence of a considerable number of gastrointestinal cancer patients harboring suicidal ideation^52^. A marked negative relationship was uncovered between mental health (depression) and suicidal ideation among these patients. However, extending past findings, our study emphasized the intricate roles of family burden, internet information utilization, and emotional support in influencing these outcomes.

The current research echoes the results of the population-based study from England, which depicted an elevated risk of suicide among cancer patients, especially during the first six months following diagnosis^53^. This points towards the critical need for timely psychological support following diagnosis. Our study's findings regarding the beneficial role of emotional support in preserving mental health among cancer patients reinforce the call for improved psychological support underscored in prior work^53^. Additionally, our investigation revealed that factors such as reduced family burden also play a pivotal role in mitigating psychological distress (depression) and suicidal ideation, thereby identifying an area that necessitates attention in cancer care.

When examining the implications of family burden in our study, we found a profound connection with the mental well-being of patients with gastrointestinal cancers. Our results reveal that as the family burden intensifies, there's a consequent detrimental effect on the patient's mental health (depression). This connection resonates with findings from another study that focused on family caregivers of colorectal cancer patients^54^. In that research, heightened family burden was directly linked to the caregivers' psychological distress. Hence, the cumulative family burden not only signals the mental strain on caregivers but also casts a shadow on the patients' psychological health. The intertwined relationship between the psychological health of patients and their caregivers underscores the mutual emotional dependencies and shared challenges they face during the cancer journey. Such dynamics suggest that the family's collective emotional health, in the context of cancer care, can't be compartmentalized, with one member's distress inevitably affecting the other. This interdependence highlights the urgency for healthcare strategies that don't just focus on the patient in isolation but instead consider the holistic psychological environment of the family. Interventions that provide support to both the patient and the caregivers can potentially create a more nurturing environment, fostering better mental health outcomes for everyone involved.

In our study, while we identified an association between the use of the internet for gathering information related to GIC and the mental health (depression) of participants, it is imperative to note that we did not delve into assessing the credibility of the sources our participants accessed. This distinction holds importance. As discussed in other studies, the trustworthiness and credibility of online health information play a critical role in shaping a patient's mental and emotional responses^55^. Encountering misleading or inaccurate information can exacerbate anxiety and distress. However, our study stops short of establishing this direct link, focusing only on the broad behavior of online health information-seeking. Nevertheless, the broader landscape of online health information underscores potential risks. If patients are navigating a maze of misinformation or misinterpreting credible sources, their mental well-being could be compromised. This becomes especially relevant for GIC patients, who, in their quest for understanding their diagnosis better, might inadvertently stumble upon non-reliable sources or misinterpret information that could amplify their fears and anxieties. Beyond traditional patient care, there is an emerging responsibility to guide patients towards credible online health resources and simultaneously equipping them with the skills to discern reliable information.

In our multigroup analysis, as detailed in Table 6, we sought to decipher the nuanced effects of GIC treatment completion status on emotional support and resultant mental health (depression). Notably, participants who had completed their GIC treatment (Group 1) demonstrated a significantly stronger correlation between emotional support and mental health (depression) compared to their counterparts who were still undergoing treatment (Group 2). This disparity might stem from the challenges post-treatment patients face; having completed their treatment regimen, they may feel isolated without the consistent care of their medical team. Moreover, as they grapple with the physical aftermath of intensive treatments and the emotional strain of navigating their return to "normal" life, the need for emotional support becomes even more paramount. This is compounded by feelings of vulnerability and potential struggles to reintegrate into their pre-cancer routines and social roles. On the other hand, those undergoing treatment, while still greatly benefiting from emotional support, might find some comfort in the regularity of their clinical care and the immediate presence of a healthcare team. Thus, while the relative influence of emotional support varies between these two groups, it remains a vital factor in bolstering mental health throughout the GIC journey, from diagnosis to recovery^56^.

Our study also showed that gender and age significantly influenced mental health (depression) and suicidal ideation. This suggests that demographic factors are important to consider in the development of personalized interventions. Acknowledging this premise, a study highlighted the prevalence of mixed anxiety and depression symptoms across different cancer types, with age and gender playing a significant role in these manifestations^57^. Another study explored how age influences the development of depressive symptoms and hopelessness, both of which are directly related to suicidal ideation^58^. Unlike our finding, where gender had no significant moderating impact, a study reported notable differences in suicidal behavior between adolescent and young adult males and females, underscoring the moderating role of gender^59^. Adding to the novelty of this study, our analysis captured a significant moderating effect of age on the relationship between mental health (depression) and suicidal ideation, indicating that this relationship may vary across different age groups. Therefore, age-specific strategies in mental health interventions may be necessary for effective suicide prevention.

The principal strength of our research rests within its methodological rigor and the comprehensive nature of its inquiry into the influences affecting depression and suicidal ideation in patients diagnosed with gastrointestinal cancer. Leveraging the PLS-SEM approach, our study uniquely examined not just the direct, but also the indirect and moderating effects of a myriad of psychosocial and demographic factors. This offered a nuanced understanding of the complex variable interactions impacting this patient population. Furthermore, our sample was carefully chosen to include both patients currently undergoing their gastrointestinal cancer treatment and those who had already completed their treatment. This heterogeneity enhanced the breadth of our insights and increased the generalizability of our conclusions. Our model integrated several control variables including education, gender, income, household occupants, and age. This approach deepened the analysis and strengthened the reliability of our findings. We further ensured the credibility of our research by employing established and recognized measures for each construct under investigation.

It is essential to acknowledge the limitations of our study. First, our study was cross-sectional in nature, preventing us from drawing definitive causal relationships between emotional support and mental health (depression). Second, the results of our study may be subject to response bias. Our participants might have reported higher levels of emotional support or improved mental health due to social desirability bias, which could potentially skew the observed associations. Third, the differential impact of emotional support based on treatment completion status was assessed based on two groups at distinct treatment stages. However, individual variability in treatment experiences, recovery rates, and personal coping mechanisms may affect the generalizability of our findings. Future research would benefit from considering these individual-level factors to achieve a more nuanced understanding of the relationships investigated. Fourth, our findings are primarily relevant to the specific population studied. The degree to which these results can be generalized to other cultural, geographical, or socioeconomic contexts is uncertain. Lastly, one limitation is the use of single-item measures for constructs such as family burden, internet information utilization, and emotional support. Although these items were designed to be concise and reduce participant burden, they might not capture the multi-faceted nature of these constructs as comprehensively as established multi-item scales. Thus, interpretations drawn from these single-item measures should be made with caution.

In conclusion, this study highlights the significant influence of psychosocial and demographic factors on mental health and suicidal ideation in patients with gastrointestinal cancer. The findings reinforce the need for comprehensive and personalized treatment plans, emphasizing mental health improvement as a crucial component of suicide prevention. It also encourages continued research to deepen our understanding and enhance our strategies in this important area of public health. We also emphasize the need for more granular studies that consider the unique challenges posed by each specific cancer type, progression, and severity.

## Data Availability

The datasets generated during and/or analyzed during the current study are available from the corresponding author on reasonable request.
